# Stroke medicine terminology: imprecise, wordy, and misleading

**DOI:** 10.1007/s00234-021-02715-w

**Published:** 2021-04-14

**Authors:** Rüdiger von Kummer, Lisa S. Babinec

**Affiliations:** 1 Institute of Diagnostic and Interventional Neuroradiology, Universitätsklinikum Dresden, Fetscherstr. 74, 01307 Dresden, Germany; 2 Seattle, USA

About 2400 years ago, Hippocrates coined the term “ἀποπληξία” (stroke) for the sudden impairment of cerebral functions and subsequent deficits. Since then, we continue to use this term for a syndrome that has a broad variety of etiologies, is triggered by various brain pathologies, and bears a high risk of permanent disability and death. It is questionable whether the term “stroke” is advantageous for the development of effective treatment [1]. The differentiation between brain ischemia and hemorrhage by brain tissue computed tomography (CT) and the identification of arterial disease by digital subtraction and CT angiography finally enabled effective ischemic stroke treatment and prophylaxis. Nevertheless, current stroke terminology still reveals uncertainty when describing imaging findings in ischemic stroke patients. In this commentary, we reflect on the imprecise, wordy, and misleading terms used in stroke diagnostics and treatment and recommend more precise terminology.

## “Acute ischemic stroke” diagnostics

The term “acute ischemic stroke” (AIS) is widely used in the scientific literature. In fact, the updated American Heart Association (AHA) guidelines (2018) for early management of acute ischemic stroke mention “acute ischemic stroke” with the variations “acute stroke,” “acute arterial ischemic stroke,” or “acute stroke syndrome” a total of 154 times, even though stroke is “acute” by definition and “ischemic stroke” is used interchangeably with “acute ischemic stroke” — a syndrome with sudden onset likely caused by ischemia [2]. “Acute ischemic stroke” is a pleonasm and reflects inaccuracy. The treatment of “AIS” as a pathological entity is more problematic however. In the literature, authors are often unclear regarding causative pathology and, as a result, one may come across terms such as “stroke imaging” and “stroke volume.” However, a clinical syndrome like headache or stroke can only be reported: it cannot be imaged, has no volume, and cannot be treated effectively.

The AHA guidelines state that “DW-MRI is more sensitive than CT for detecting AIS^70,71^” (e389) [2]. This guideline begs the question: How were CT and DWI sensitivities for the syndrome “stroke” assessed? What reference standard was used to assess diagnostic accuracy of CT and DWI for this clinical syndrome? Unfortunately, both references (70 and 71) cited in the AHA guidelines in support of this statement did not in fact determine CT and DWI sensitivity for stroke pathology; instead, these studies evaluated the impact of clinical baseline variables on clinical outcomes.

The diagnosis of specific brain tissue and vessel pathology in the individual patient is a precondition for effective treatment of ischemic stroke. Yet, terminology describing findings on CT and MRI remains remarkably imprecise, wordy, and misleading. For example, “early ischemic changes, signs of ischemia, loss of the insular ribbon, obscuration of the lentiform nucleus, loss of gray-white matter distinction, ischemic core or infarct core, hypodensity, hyperdense middle cerebral artery sign, and fluid-attenuated inversion recovery vascular hyperintensity” are frequently used to describe nothing more than focal gray matter hypoattenuation caused by ischemic brain tissue water uptake or cerebral artery thrombo-embolic obstruction. Because ion depletion in the intercellular space drives net water uptake in ischemic brain tissue, it has been labeled “ionic edema” [3]. Recognition of ionic edema is relevant, because it indicates that the brain tissue has suffered (or still suffers) from severe ischemia which cannot be tolerated for more than 30 min [4]. As such, when compared to the phrases mentioned above, it is more appropriate and informative to describe localization and extent of ionic edema on CT of the ischemic brain. In contrast to CT, diffusion-weighted MRI (DWI) is sensitive to proton diffusion impairment due to a shift of ions and water from the extracellular space into the intracellular space with subsequent cell swelling (cellular or cytotoxic edema) but without net water uptake and thus not affecting X-ray attenuation [5]. The term “cytotoxic” is problematic as well, because toxins are not involved here. Hence, describing cellular edema as “DWI-lesion,” “DWI signal hyperintensity,” or “brain infarction” is both vague and misleading. Cellular edema is triggered by ion pump failure due to cerebral blood flow impairment that may allow functional recovery with blood flow restoration [6]. This means that both types of ischemic brain edema — ionic and cellular — are triggered by different levels of ischemia which have individual predictive values for brain tissue survival.

The terms “ischemic core” or “infarct core” are also imprecise and misleading, because they describe something that is in the middle of something else. What, one may ask, is in the middle of an infarction? If “ischemic core” refers to the arterial territory with critical low blood flow values, it is inaccurate to assume that this territory is always completely surrounded by other tissues with better flow. If, however, “core” describes irreversibly injured brain tissue, “infarction” is a more accurate term. Additionally, given that CT or MRI perfusion imaging directly detects and measures brain ischemia, the term “CT perfusion” applied for CT perfusion imaging (CTP) incorrectly suggests circulation within the CT scanner. Likewise, the term “perfusion-weighted imaging” (PWI) is also misleading. The maps derived from cerebral contrast flow dynamics display contrast transit times, cerebral blood flow and volume represent numbers, not weighted contrast that is found in DWI where proton diffusion is only one component of image contrast.

A meta-analysis of seven randomized controlled thrombectomy trials provides evidence that even ischemic stroke patients with “large infarctions at baseline” may benefit from thrombectomy [7]. Despite this evidence, the assessment and measurement of ischemic infarction soon after stroke onset was recently deemed crucial when treating ischemic stroke patients [8]. The authors even developed an automated machine learning approach to detect and quantify infarction on CT within 6 h of symptom onset using DWI as reference standard, though neither CT nor DWI display “infarction” soon after stroke onset. Moreover, it has previously been shown that histological changes indicating irreversible injury are barely detectable under the microscope after experimental middle cerebral artery occlusion and affect less than 20% of neurons within the first 6 h of ischemia [9]. It is unlikely, therefore, that ionic edema detected by CT or cellular edema detected by DWI represent “infarction” or “infarct core.” Thus, early CT and DWI findings after ischemic stroke should not be used to develop algorithms that falsely identify stroke patients who categorically will not benefit from thrombectomy. Finally, because neither modality shows brain infarction, early CT or MRI is an inappropriate baseline comparator for assessing “infarct growth.”

## “Acute stroke” treatment

Effective treatment of ischemic stroke patients primarily involves treatment of the causative arterial pathology which includes occlusive thrombosis or embolism in cerebral arteries. Thromb-emboli can be lysed with thrombolytics or extracted by thrombectomy with special devices. The oft used phrase — “treatment with thrombolysis” — is ill-defined and euphemistic because it suggests that thrombolysis can be achieved in each patient. Nonetheless, we treat with thrombolytics — not with thrombolysis — hoping that the thrombus will be lysed, which is the case in less than 40% of ischemic stroke patients.

Thrombectomy is more effective. Because non-mechanical or extravascular thrombectomy do not exist, the terms “mechanical thrombectomy (MT)” and “endovascular thrombectomy” are also pleonasms. “Thrombectomy” is clear enough in describing the extraction of thrombotic material from cerebral arteries using endovascular devices. The recent AHA guidelines use the term “mechanical thrombectomy” 79 times with odd variations of “mechanical thrombectomy eligibility” and “pre-mechanical thrombectomy era” [2]. Additionally, the guidelines mention a “mechanical closure” of patent foramen ovale as well, begging the question, what is non-mechanical closure? [2].

The dilemma of brain image reading, interpretation, and reporting in ischemic stroke is that it lacks a clear reference standard, most likely because autopsies are rarely performed soon after brain imaging in clinical medicine for obvious reasons. The study of events and repeated imaging during follow-up can determine the predictive value of baseline imaging findings, but not sensitivity and specificity for baseline brain pathology. Image interpretation should respect the physics of image generation that is relatively simple for CT, but far more complex for MRI. Findings should be described in terms of the most likely brain pathology and avoid imprecise terms such as “signs” or “core” as well as any overinterpretation such as mistaking edema for infarction.
